# Exposure to High Concentrations of Tetrabromobisphenol A Slows the Process of Tissue Regeneration and Induces an Imbalance of Metabolic Homeostasis in the Regenerated Intestines of *Apostichopus japonicus*

**DOI:** 10.3390/genes15111448

**Published:** 2024-11-09

**Authors:** Zi Wang, Xiaojun Song, Wenhui Yin, Kuntao Shi, Ying Lin, Jixiang Liu, Xiaohan Li, Jiabo Tan, Junjie Rong, Kefeng Xu, Guodong Wang

**Affiliations:** 1 School of Marine Science and Engineering, Qingdao Agricultural University, Qingdao 266109, China; m17860753620@163.com (Z.W.); songxiaojun@qau.edu.cn (X.S.); 15066795631@163.com (W.Y.); m17852153793@163.com (Y.L.); 17865322108@163.com (J.L.); li17685746186@163.com (X.L.); jiabo@qau.edu.cn (J.T.); 18763330296@163.com (J.R.); 2 Weihai Huancui District Marine Development Research Center, Weihai 264200, China; shikuntao@163.com; 3 Marine Science Research Institute of Shandong Province, National Oceanographic Center, Qingdao 266104, China

**Keywords:** *Apostichopus japonicus*, tissue regeneration, lipid metabolism, gut microbiome, TBBPA

## Abstract

Background: Tissue regenerative capacity following evisceration, potentially influenced by environmental contaminants and intestinal microflora, is essential for the financial success of *Apostichopus japonicus* farming. However, the morphological structure, gut microbiome composition, and genes expression pattern of the regenerated gut after exposure to high levels of TBBPA remain poorly unclear. Methods: In this research, the effect of TBBPA exposure on tissue regeneration in *A. japonicus* was investigated through a comprehensive multi-omics approach. Results: Our results showed that the integrity, the intestinal wall thickness, and the villi length of the regenerated intestines in *A. japonicus* decreased after treatment with high levels of TBBPA. The findings from PCoA and NMDS analyses revealed that the microbial community composition was significantly altered following exposure to high concentrations of TBBPA in the regenerated intestines of *A. japonicus*. The KEGG pathway enrichment analysis indicated that the DEGs (differentially expressed genes) were predominantly enriched on metabolism and immunity-related signaling pathways after exposure to high levels of TBBPA. These included pathways involved in the PPAR signaling pathway, ECM receptor interaction, glycerolipid metabolism, and fatty acid degradation. Interestingly, the results have demonstrated that there are 77 transcript factors that were significantly different after exposure to TBBPA. Conclusions: These results suggested that high levels of exposure to TBBPA induces an imbalance of the metabolic homeostasis by regulating the expression levels of transcription factors in the regenerated intestines of *A. japonicus*.

## 1. Introduction

Regeneration of injured or lost tissue/organs is an intriguing process in which numerous cellular events occur to form the new tissue/organs [1]. There are two hypotheses to explain why some animals have the ability of tissue regeneration and others have not. One hypothesis is that tissue regeneration is a fundamental ability in all animals, and that many species have lost the ability of tissue regeneration during their evolutionary history, as the ability of tissue regeneration may have been detrimental or simply neutral with regard to survival in certain animals. The alternative hypothesis is that the ability of tissue regeneration was independently selected for each example in evolutionary history, and arose separately as an adaptation to environmental pressures [2,3,4]. In any case, the ability of tissue regeneration is affected by many factors, such as environmental toxins, gut microbes, probiotics, diet, and PAMP (pathogenic-associated molecular patterns) [5,6,7,8].

Recent studies have demonstrated that gut microbes affect the healing and regeneration of intestinal epithelial cells, nerves, liver, and bone [8,9,10,11]. The probiotic *Lactobacillus reuteri* can promote the process of intestinal regeneration through the wingless type (Wnt) signaling pathway, indicating that probiotic treatment could be used to enhance intestinal regeneration as a nutritional strategy [7,12]. The lactate can promote tissue regeneration in a Wnt3/β-catenin–dependent manner through being recognized by GPCR (G-protein-coupled receptor) on Paneth and stromal cells [13]. Additionally, the administration of lactate protected the mice from exposure to chemotherapy- or radiation-induced intestinal damage [13]. Enhancing the regenerative capacity of sensory neurons can be achieved through various environmental stimuli, such as physical activity and cognitive challenges [14]. For instance, research has shown that periodic fasting can encourage the regrowth of axons in mice after injury to the sciatic nerve. This axonal recovery is linked to the synthesis of the microbial byproduct indole-3-propionic acid (IPA) by certain beneficial bacteria in the gut, particularly *Clostridium sporogenes*, which are a Gram-positive species.

The process of tissue regeneration requires precise genetic regulation. Recent studies have demonstrated that Wnt, Hippo, and PPAR signaling pathways were involved in regulating the intestinal regeneration process [15,16,17,18]. Interestingly, CL429, an innovative targeting ligand, serves as a ligand for both Toll-like receptor 2 (TLR2) and nucleotide-binding oligomerization domain 2 (NOD2) to regulate the regeneration of intestinal stem cells (ISCs) by activating the Hippo and Wnt signaling pathways in mice. This finding indicates a significant interplay between the immune system and the process of tissue regeneration [19,20]. In addition, inflammation and metabolism also play vital roles in the process of tissue regeneration [21,22].

The sea cucumber (*A. japonicus*), a marine species within the phylum Echinodermata and class Holothuroidea, holds considerable commercial importance in East Asian markets, renowned for its nutritional richness [23]. Because of their remarkable ability to undergo evisceration and subsequent tissue regeneration, sea cucumbers are increasingly being recognized as ideal models for investigating tissue regeneration [24]. TBBPA (Tetrabromobisphenol A), a potent and dependable BFR (brominated flame retardant), has been detected across various biotic and abiotic environments, encompassing sediments, aquatic systems (both freshwater and marine), terrestrial soils, human biological specimens, indoor dust particles, and notably, within human milk specimens [25,26]. Extensive research has demonstrated that TBBPA has an apparently toxic effect on growth and development, disrupts the endocrine system, increases oxidative stress, damages the reproductive organ, and induces neurodevelopmental and cardiac toxicity [6,27,28,29,30,31]. As a result, the potential risks of TBBPA on tissue regeneration have caused concern among toxicologists. However, the morphological structure, gut microbiome composition, and expression regulation of the regenerated gut in *A. japonicus* after exposure to high levels of TBBPA remain poorly unclear. This research endeavored to investigate the correlation between TBBPA exposure and the intestinal regeneration in *A. japonicus*. These results found that exposure to high concentrations of TBBPA slows the process of tissue regeneration and induces an imbalance of metabolic homeostasis in the regenerated intestine of *A. japonicus*.

## 2. Materials and Methods

### 2.1. Ethics Statement

All animal subjects utilized in the experimental protocols were managed in compliance with the ethical standards set forth by the School of Marine Science and Engineering at Qingdao Agricultural University, aligning with national regulations governing the care and utilization of laboratory animals.

### 2.2. Experimental Animals

Healthy sea cucumbers (*A. japonicus*) were procured from a farm of sea cucumber aquaculture at Qingdao, China, with individual weights averaging 50.0 ± 5.0 g. The sea cucumbers were acclimated to the laboratory conditions for a period of 1 week in a recirculating aquaculture system, which was maintained at a pH level of 7.8 to 8.2, a temperature range of 15 to 17 °C, and a salinity level of 28 to 30‰. Following the acclimation period, evisceration in the sea cucumbers was induced by injecting 2 mL of a 0.35 M potassium chloride (KCl) solution into their body cavities. Post-evisceration, the animals were randomly allocated into 12 tanks, each measuring 300 × 500 × 600 mm, with a stocking density of 25 individuals per tank to standardize the experimental setup.

### 2.3. TBBPA Exposure Experiment

The eviscerated *A. japonicus* were then cultured in seawater containing TBBPA at levels of 0, 3, 5, and 7 nmol/L, with dimethyl sulfoxide (DMSO) serving as a cosolvent. After an 11-day exposure period to TBBPA, the coelomic fluid was collected from *A. japonicus* for enzyme activity analysis. Additionally, the regenerated intestinal tissues were excised for 16S rDNA gene sequencing and RNA transcriptome profiling. Post-collection, all samples were rapidly frozen in liquid nitrogen and subsequently stored at −80 °C for further analysis.

### 2.4. Enzyme Activity Analysis

The coelomic fluid samples were taken from the −80 °C freezer and thawed on ice. Subsequently, the coelomic fluid samples were subjected to centrifugation for a duration of 20 min at 5000× *g* at 4 °C. The supernatant of coelomic fluid samples was obtained, and was used for examining the enzyme activity of coelomic fluid samples within 24 h. The activities of lysozyme (LZM), superoxide dismutase (SOD), and alkaline phosphatase (AKP) in the samples were measured using commercial assay kits obtained from Jiancheng Biotechnology Co., Nanjing, China. The procedures were carried out in accordance with the manufacturer’s recommended protocols.

### 2.5. Genomic DNA Extraction and 16S rDNA Gene Sequencing

Genomic DNA from the regenerated intestines was extracted using commercial kits and subsequently assessed via 1.0% agarose gel electrophoresis. The V4-V5 regions of the 16S rDNA gene were targeted for amplification and high-throughput sequencing using the commercially available barcoded fusion primers 338F: 5′-ACTCCTACGGGAGGCAGCA-3′ and 806R: 5′-GGACTACHVGGGTWTCTAAT-3′. The PCR amplification process for the 16S rDNA gene involved an initial denaturation at 95 °C for 30 s, followed by 25 cycles of denaturation at 95 °C for 10 s, annealing at 53 °C for 30 s, and extension at 72 °C for 30 s. Equal molar concentrations of the PCR products were pooled and sequenced on the Illumina MiSeq platform by Biomarker Technologies Co., Ltd (Beijing, China), and the raw data were submitted to the China National Center for Bioinformation (CNCB, https://www.cncb.ac.cn/?lang=en, accessed on 1 November 2024) under the accession numbers of CRA020120.

### 2.6. The Diversity Analysis of Intestinal Microbiota

The Trimmomatic 0.33 software was used for quality filtering, and Cutadapt 1.9.1 was used to recognize and cut the primer sequences. For merging paired-end sequence reads, the USEARCH 10 software was used. Subsequently, the Uparse 11 software was used to analyze the clean reads of high-quality sequences. The USEARCH 10 software was used for the operational classification unit (OTU) cluster, and the sequences were assigned to the same OTU if the similarity was greater than or equal to 97%. The RDP Classifier 2.2 software was used for annotating the information of classification. MSA (multiple sequence alignment analysis) was conducted using the MAFFT version 7.2 software. The phylogenetic relationships of OTUs and dominant species were constructed through the MEGA X software. The alpha diversity index including the Shannon, Chao1, ACE, and Simpson richness estimator was analyzed by the QIIME 2 software. The beta diversity reflected by NMDS (non-metric multidimensional scaling) or PCoA (principal coordinate analysis) was analyzed using the QIIME2 software. The analysis of taxonomic abundance using the QIIME2 software and R language was used for visualizing.

### 2.7. RNA Extraction, Library Preparation, and Digital Gene Expression (DGE) Sequencing

Total RNA was extracted from the samples using the Trizol Reagent (Thermo Fisher Scientific, Franklin, MA, USA). Following extraction, the RNA samples were assessed for purity, concentration, and integrity using a NanoDrop 2000 spectrophotometer (Thermo Fisher Scientific, Franklin, MA, USA) and the Agilent Bioanalyzer 2100 system (Agilent Technologies, Santa Clara, CA, USA).). RNA sequencing libraries were prepared using the Hieff NGS Ultima Dual-mode mRNA Library Prep Kit for Illumina, as per the guidelines provided by Yeasen Biotechnology (Shanghai) Co., Ltd. (Shanghai, China). The sequencing libraries were utilized for generating 2 × 150 bp paired-end reads on an Illumina NovaSeq platform, and the raw data were submitted to the China National Center for Bioinformation (CNCB, https://www.cncb.ac.cn/?lang=en, accessed on 4 November 2024) under the accession numbers of CRA020139. To ensure the quality of the data, the raw sequences were processed to remove adapters, low-quality reads, and other contaminants. The resulting clean reads were aligned to the *A. japonicus* reference genome, which can be accessed at the NCBI Genome database (https://www.ncbi.nlm.nih.gov/genome/12044, accessed on 6 November 2017). Transcripts, both known and novel, were identified using the RABT (StringTie Reference Annotation Based Transcript) assembly method. Gene function annotation was conducted by aligning the sequences against various databases, including Pfam (Protein family), NR (NCBI non-redundant protein sequences), GO (Gene Ontology), KOG/COG (Clusters of Orthologous Groups of proteins), Swiss-Prot, and KO (KEGG Ortholog database), to infer potential functions and biological roles. Gene expression levels were estimated using the FPKM (fragments per kilobase of transcript per million fragments mapped) method, which normalizes the gene expression data based on the transcript length and the total number of reads mapped. Differential gene expression analysis was conducted to identify DEGs among different groups using the DESeq 2 software, with a threshold of an adjusted *p*-value ≤ 0.05 and a |Fold-Change (FC)| ≥ 2.0.

### 2.8. Gene Ontology (GO) and Kyoto Encyclopedia of Genes and Genomes (KEGG) Pathway Enrichment Analyses

For the Gene Ontology (GO) enrichment analysis, the Wallenius non-central hyper-geometric distribution provided by the GOseq R package was utilized, with an adjusted *p*-value ≤ 0.05. Furthermore, the KOBAS 2.0 software was employed to perform KEGG pathway enrichment analyses on the differentially expressed genes (DEGs). Pathways with an adjusted *p*-value ≤ 0.05 were considered to be significantly enriched. The heatmaps and clustering analysis were conducted utilizing the BMKCloud platform (www.Biocloud.net, accessed on 22 February 2023), employing the Bray–Curtis method for distance calculation and the average linkage method for hierarchical clustering.

### 2.9. Statistical Analysis

The one-way analysis of variance (ANOVA) was conducted to assess differences in the intestinal wall thickness, villi length, enzymatic activities, bacterial diversity of the regenerated intestines, and gene expression levels across various treatment groups. The homogeneity of variance assumption is verified prior to ANOVA to ensure the validity of the test results. If significant differences were indicated by ANOVA, the least significant difference (LSD) method was employed for post hoc pairwise comparisons to identify which groups differed from each other. All data were presented as means ± SD (standard deviation) unless otherwise noted.

## 3. Results

### 3.1. High Levels of Exposure to TBBPA Can Slow the Process of Intestine Egeneration

**Figure 1:**
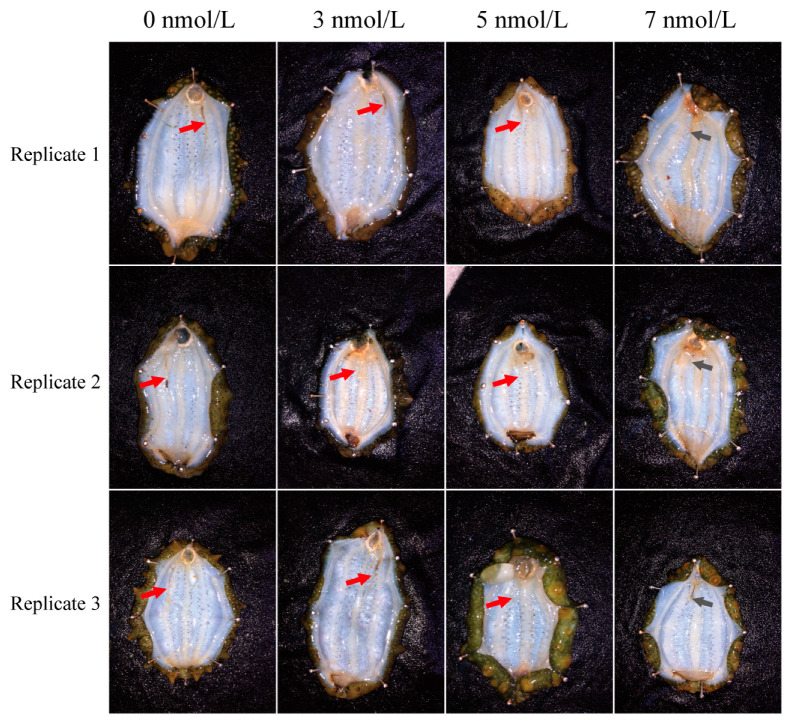
Morphological structure of the regenerated intestines in *A. japonicus* after exposure to different levels of TBBPA. There are three replications for each level. Red arrows indicate the regenerated intestines, gray arrows indicate the wound for evisceration.

The results showed that exposure to high levels of TBBPA slowed the percentage of intact regenerated intestines in the *A. japonicus* (Figure 1 and Table 1). The percentage of intact regenerated intestines was 100% after 11 days of exposure to TBBPA at 0 and 3 nmol/L, and 32% and 0% after 11 days of exposure to TBBPA at 5 and 7 nmol/L, respectively (Table 1).

**Figure 2:**
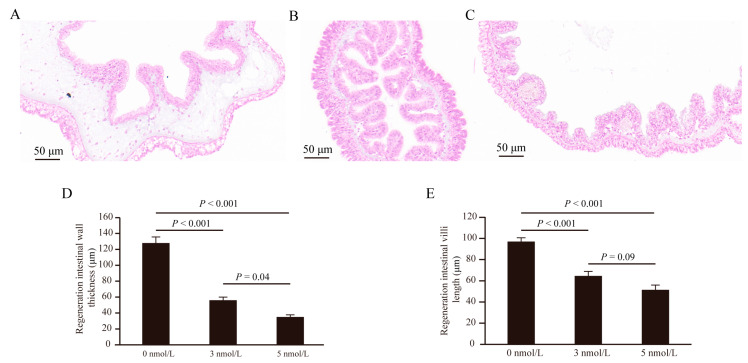
The intestinal wall thickness and the villi length of the regenerated intestine decreased after 11 days of exposure to different levels of TBBPA. (**A**–**C**) The effects of TBBPA exposure on the regenerated intestine were examined at concentrations of 0 nmol/L (**A**), 3 nmol/L (**B**), and 5 nmol/L (**C**) using hematoxylin-eosin (HE) staining. (**D**) The intestinal wall thickness of the regenerated intestines was measured following exposure to TBBPA at concentrations of 0 nmol/L, 3 nmol/L, and 5 nmol/L. (**E**) The villi length of the regenerated intestines was measured following exposure to TBBPA at concentrations of 0 nmol/L, 3 nmol/L, and 5 nmol/L.

Interestingly, the intestinal wall thickness and the villi length of the regenerated intestine were affected after exposure to TBBPA (Figure 2). The thickness of the regenerated intestinal wall decreased after exposure to 3 and 5 nmol/L TBBPA (*p* < 0.001) (Figure 2A–D). The villi length of the regenerated intestine decreased after exposure to 3 and 5 nmol/L TBBPA (*p* < 0.001) (Figure 2).

### 3.2. Enzyme Activities in Antioxidant and Immune Systems

**Figure 3:**
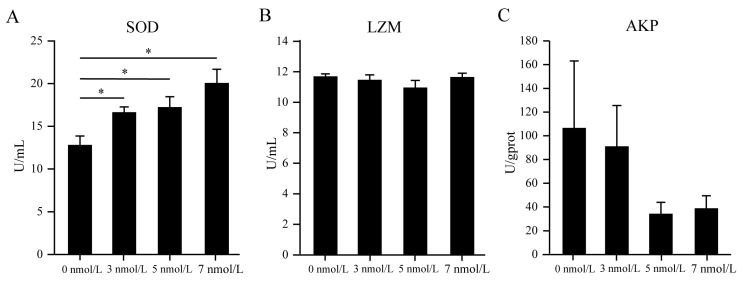
The coelomic fluid enzymatic activity of SOD (**A**), LZM (**B**), and AKP (**C**) in *A. japonicus* after exposure to TBBPA. * Presents *p* < 0.05.

In the coelomic fluid of *A. japonicus*, the enzyme activity assays indicated an increase in superoxide dismutase (SOD) levels, whereas no significant changes were observed in lysozyme (LZM) levels after an 11-day exposure to TBBPA at concentrations of 3, 5, and 7 nmol/L (Figure 3A,B). Nevertheless, a decrease in alkaline phosphatase (AKP) levels was observed; however, this reduction was not statistically discernible (Figure 3C). These findings suggested that TBBPA exposure affects the enzyme activity in the coelomic fluid of *A. japonicus*.

### 3.3. High Levels of TBBPA Exposure Affects the Microbial Community of the Regenerated Intestines in A. japonicus

**Figure 4:**
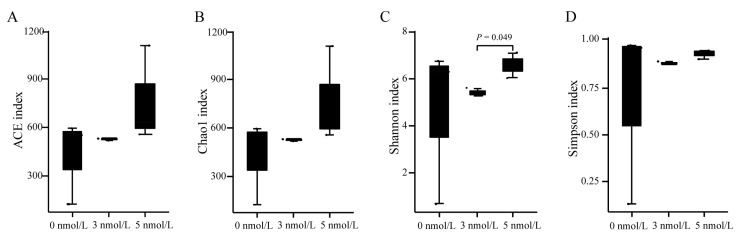
The alpha diversity indexes of gut microbiome in the regenerated intestines of *A. japonicus* after exposure to different levels of TBBPA. (**A**) ACE index, (**B**) Chao1 index, (**C**) Shannon index, and (**D**) Simpson index of gut microbiome in the regenerated intestines of *A. japonicus* after exposure to different levels of TBBPA.

**Figure 5:**
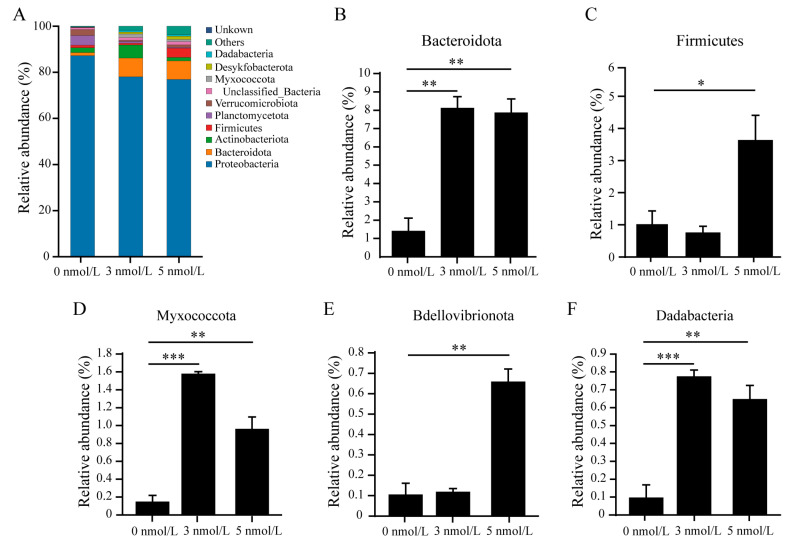
The gut microbiome composition at the phylum level in the regenerated intestines of *A. japonicus* was evaluated after exposure to TBBPA. (**A**) The microbial community composition at the phylum level in the regenerated intestines of *A. japonicus* after exposure to TBBPA. (**B**–**F**) The relative abundance of Bacteroidota (**B**), Firmicutes (**C**), Myxococcota (**D**), Bdellovibrionota (**E**), and Dadabacteria (**F**) after exposure to TBBPA. * Means *p* < 0.05; ** means *p* < 0.01; *** means *p* < 0.001.

Analysis of the diversity of the regenerated gut microbiome after 11 days of exposure to TBBPA revealed that high levels of exposure to TBBPA affects the microbial community of the regenerated intestines in *A. japonicus*. Alpha diversity analysis found no significant difference in ACE, Chao1, and Simpson diversity indices, while the Shannon diversity index increased after 11 days of TBBPA exposure (Figure 4). The microbial community analysis within the regenerated intestines indicated that the predominant bacterial phyla were Proteobacteria (87.21%), Planctomycetes (4.20%), Verrucomicrobiota (2.59%), and Actinobacteriota (2.12%) in the control group without TBBPA exposure. In contrast, the groups exposed to 3 nmol/L TBBPA exhibited a shift in composition, featuring Proteobacteria (78.04%), Bacteroidota (8.12%), and Actinobacteriota (5.70%) as the major constituents. Furthermore, the group exposed to 5 nmol/L TBBPA showed a similar trend with Proteobacteria (76.88%), Bacteroidota (8.10%), and an emergence of Firmicutes (3.99%) among the dominant species. These findings highlight the impact of TBBPA exposure on the gut microbiome, suggesting a dose-dependent alteration in microbial distribution and composition (Figure 5A). In addition, the relative abundance of Bacteroidota, Firmicutes, Myxococcota, Bdellovibrionota, and Dadabacteria increased after 11 days of TBBPA exposure (Figure 5B–F).

**Figure 6:**
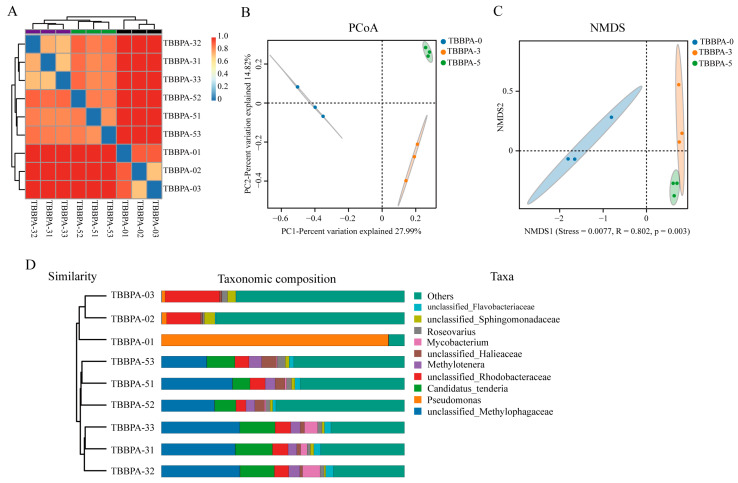
The diversity of the microbial community in the regenerated intestines of *A. japonicus* was analyzed following exposure to TBBPA. (**A**) The sample clustering heatmap analysis between the TBBPA exposure groups and the control. (**B**–**D**) The analysis of PCoA (**B**), NMDS (**C**), and taxonomic composition (**D**) demonstrated the diversity of gut microbiome in the regenerated intestines of *A. japonicus* after exposure to TBBPA. TBBPA-0, TBBPA-3, and TBBPA-5 means the 0 nmol/L TBBPA exposure group, the 3 nmol/L TBBPA exposure group, and the 5 nmol/L TBBPA exposure group, respectively.

To further explore the difference of the gut microbiome in the regenerated intestines of *A. japonicus* after exposure to TBBPA, the beta diversity analysis was performed. The sample clustering heatmap showed a significant divergence in the species distribution and composition of the gut microbiome in the regenerated intestines of *A. japonicus* between the non-TBBPA and TBBPA exposure groups (Figure 6A). The result of PCoA (principal coordinates analysis) demonstrated that the diversity of gut microbiome in the regenerated intestine of sea cucumber have not overlapped after TBBPA exposure (Figure 6B). In addition, the results of the NMDS and phylogenetic analysis of taxonomic composition have a similar phenomenon compared with the result of PCoA analysis (Figure 6C,D), suggesting that high levels of exposure to TBBPA affects the microbial composition and diversity in the regenerated intestines of *A. japonicus*.

### 3.4. High Levels of Exposure to TBBPA Induces an Imbalance of Metabolic Homeostasis in the Regenerated Intestine

**Figure 7:**
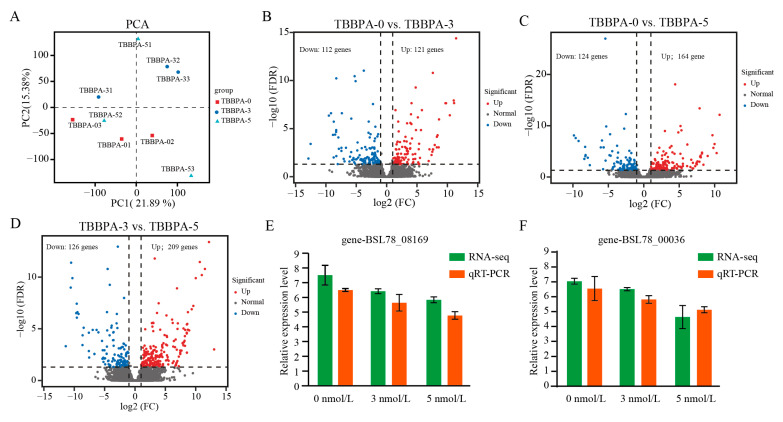
The DEGs analysis of the regenerated intestines following exposure to TBBPA. (**A**) PCA analysis revealed differences of gene expression in the regenerated intestines of *A. japonicus* between the TBBPA exposure groups and the control group. (**B**–**D**) Volcanic plots illustrate the distribution of DEGs in the regenerated gut between control and TBBPA-3 (**B**), control and TBBPA-5 (**C**), and TBBPA-3 and TBBPA-5 (**D**). TBBPA-0, TBBPA-3 and TBBPA-5 means the 0 nmol/L TBBPA exposure group, the 3 nmol/L TBBPA exposure group, and the 5 nmol/L TBBPA exposure group, respectively. (**E**,**F**) The qRT-PCR was employed to assess the expression levels of randomly selected genes.

A total of 62.13 gigabases (Gb) of clean data was obtained, with each sample exhibiting a Q30 base percentage of 94.83% or higher. The homologous alignment of pairwise sequence to the reference genome in each sample was 67.39–68.79% (Table 2). The result of PCoA (principal coordinates analysis) has indicated that the gene expression level in the regenerated intestines have not overlapped between the control group and the 3 nmol/L TBBPA exposure group, but the gene expression level in the regenerated intestines was dispersed after exposure to 5 nmol/L TBBPA (Figure 7A). Analysis of DEGs demonstrated that a total of 233 DEGs was detected between the control group and the 3 nmol/L TBBPA exposure group, with 121 upregulated genes and 112 downregulated genes (Figure 7B). Similarity, a total of 288 DEGs was detected between the 5 nmol/L TBBPA exposure group and the control group, with 164 upregulated genes and 124 downregulated genes (Figure 7C) and a total of 335 DEGs was detected between the 3 nmol/L TBBPA exposure group and the 5 nmol/L TBBPA exposure group, with 209 upregulated genes and 126 downregulated genes (Figure 7D).

**Figure 8:**
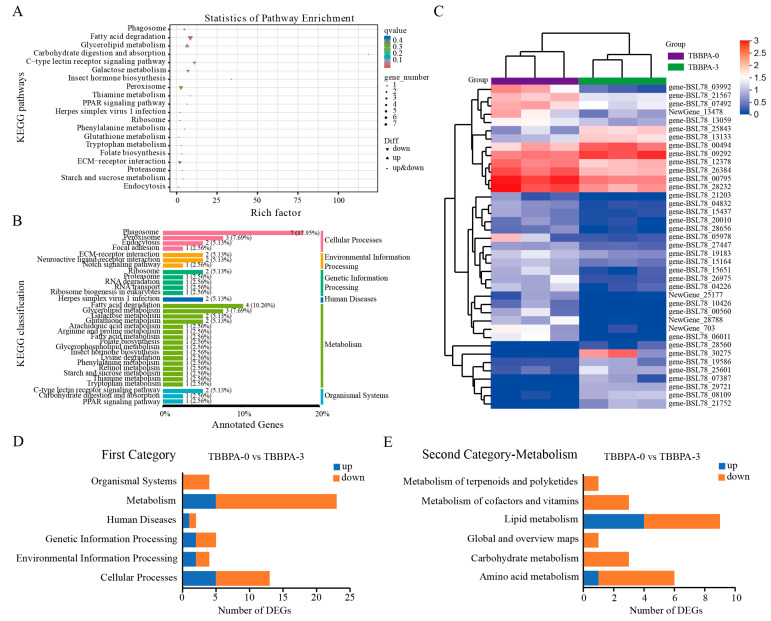
KEGG pathway enrichment analysis of DEGs in the regenerated intestines between the control (TBBPA-0) group and the TBBPA-3 group. (**A**) The top 20 pathways of KEGG pathways were revealed. (**B**) The KEGG classification and proportion of pathways. (**C**) The heatmap of DEGs in the regenerated intestines between the control and the TBBPA-3 groups. (**D**) The number of KEGG pathways in the first category in the regenerated intestines of *A. japonicus* between the control and TBBPA-3 groups. (**E**) The number of KEGG pathways for metabolism in the second category in the regenerated intestine of *A. japonicus* between the control and TBBPA-3 groups. TBBPA-0 and TBBPA-3 means the 0 nmol/L TBBPA exposure group and the 3 nmol/L TBBPA exposure group, respectively.

**Figure 9:**
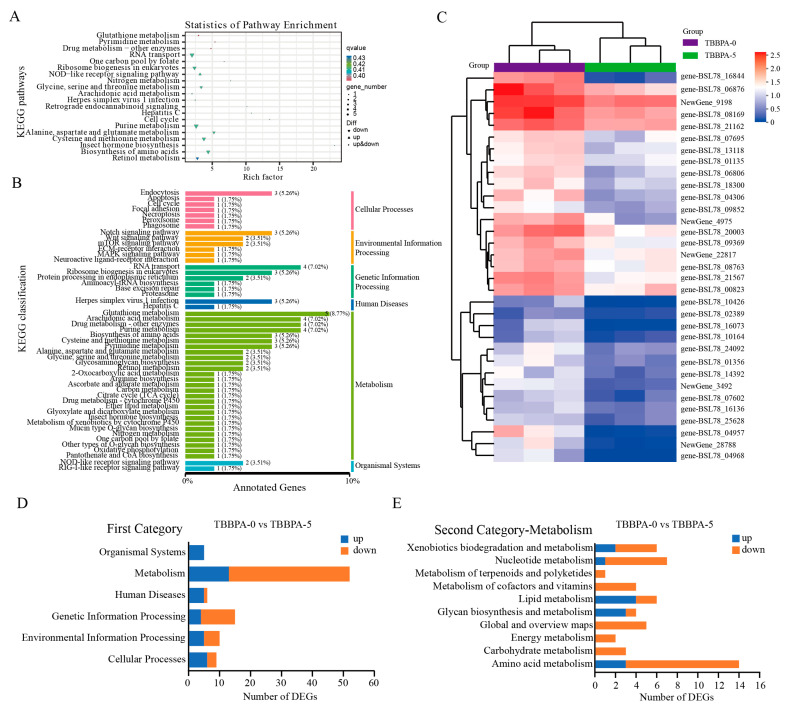
KEGG pathway enrichment analysis of DEGs in the regenerated intestines between the control (TBBPA-0) group and the TBBPA-5 group. (**A**) The top 20 pathways of KEGG pathways were revealed. (**B**) The KEGG classification and proportion of KEGG pathways. (**C**) The heatmap of DEGs in the regenerated intestines between the control group and the TBBPA-5 group. (**D**) The number of KEGG pathways in the first category in the regenerated intestine of *A. japonicus* between the control and the TBBPA-5 groups. (**E**) The number of KEGG pathways for metabolism in the second category in the regenerated intestine of *A. japonicus* between the control and the TBBPA-5 groups. TBBPA-0 and TBBPA-5 means the 0 nmol/L TBBPA exposure group and the 5 nmol/L TBBPA exposure group, respectively.

To explore the function of DEGs in gene networks and signaling pathways, the KEGG pathway enrichment analysis was conducted. The result indicated that 39 DEGs between the 3 nmol/L TBBPA exposure group and the control group were significantly enriched on metabolism- and immunity-related signaling pathways, such as the PPAR signaling pathway, fatty acid degradation, ECM receptor interaction, and glycerolipid metabolism (Figure 8). In addition, all of enriched signaling pathways were composed of six categories: environmental information processing, cellular processes, human diseases, genetic information processing, organismal systems, and metabolism (Figure 8B,D). Among them, metabolism-related pathways accounted for the highest proportion in the first category (Figure 8D,E). In the second category of metabolism, the lipid metabolism signaling pathway and amino acid metabolism accounted for the highest proportions between the control group and the 3 nmol/L TBBPA exposure group (Figure 8E). A total of 57 DEGs between the 5 nmol/L TBBPA exposure and control groups were enriched on metabolism-related signaling pathways (Figure 9). In the second category of metabolism, the amino acid metabolism, lipid metabolism, nucleotide metabolism, and xenobiotics biodegradation and metabolism signaling pathways accounted for the highest proportion between the control group and the 5 nmol/L TBBPA exposure group (Figure 9D,E).

**Figure 10:**
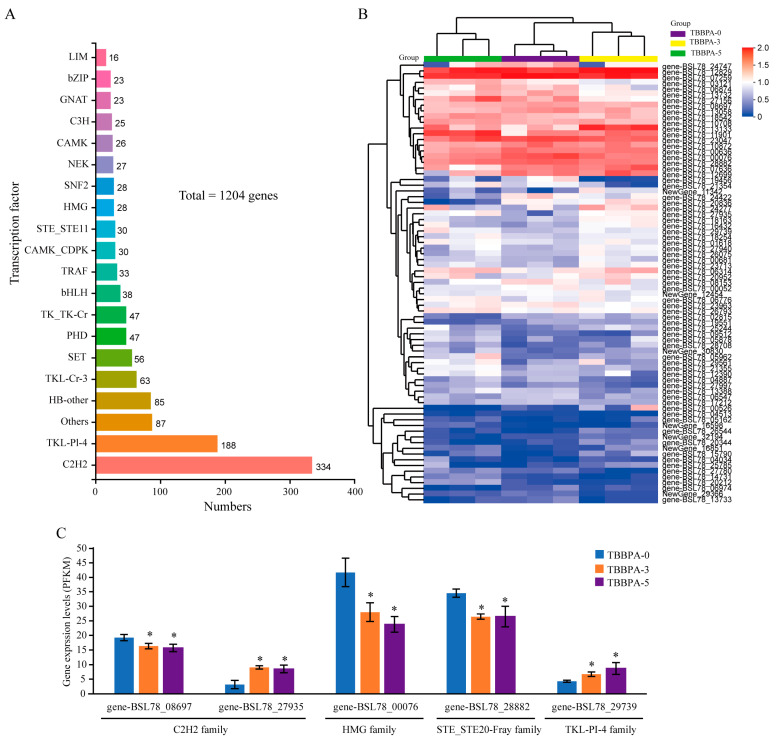
The composition and expression levels of transcription factors in the regeneration of *A. japonicus* after exposure to TBBPA. (**A**) The transcription factors found in the transcriptome of the regenerated intestines. (**B**) The DEGs of transcript factors in the regenerated intestines after exposure to TBBPA. (**C**) The expression levels of gene-BSL78_08697 (C2H2 family), gene-BSL78_00076 (HMG family), gene-BSL78_28882 (STE_STE20-Fray family), gene-BSL78_27935 (C2H2 family), and gene-BSL78_29739 (TKL-Pl-4 family) in the regeneration after exposure to TBBPA. TBBPA-0, TBBPA-3, and TBBPA-5 means the 0 nmol/L TBBPA exposure group, the 3 nmol/L TBBPA exposure group, and the 5 nmol/L TBBPA exposure group, respectively. * Means *p* < 0.05.

Gene expression tends to require regulation by transcription factors. Therefore, the composition and expression levels of transcription factors were explored. The findings revealed that the transcriptome of the regenerated intestines contained 1024 transcription factors, classified across 19 distinct gene families. Among them, the C2H2 family has 334 members (Figure 10A). The results regarding the expression levels of transcription factors indicated that a significant difference was observed for 77 transcription factors following exposure to TBBPA (Figure 10B). Interestingly, the expression level of gene-BSL78_08697 (C2H2 family), gene-BSL78_00076 (HMG family), and gene-BSL78_28882 (STE_STE20-Fray family) decreased after exposure to the 3 nmol/L and 5 nmol/L TBBPA, while the expression level of gene-BSL78_27935 (C2H2 family) and gene-BSL78_29739 (TKL-Pl-4 family) increased (Figure 10C). These results suggested that high levels of exposure to TBBPA induces an imbalance of metabolic homeostasis through regulating the expression levels of transcription factors in the regenerated intestines of *A. japonicus*.

## 4. Discussion

The capacity for regeneration is conserved throughout evolutionary history from the common ancestor of the Metazoa to mammals. However, most capacity of regeneration has been lost in the Ecdysozoa, birds, and mammals. Sea cucumbers (*A. japonicus*), as the ancestor of Deuterostomia, are able to regenerate complex organs, such as the digestive tract, respiratory tree, muscle, nerve cord, and tentacles [1,32]. Recent research indicates that tissue regenerative capabilities can be influenced by a variety of factors, including environmental toxins, gut microbiota, and probiotic substances [5,6,7,8]. In this study, we found that exposure to high concentrations of TBBPA slows the process of tissue regeneration and induces an imbalance of metabolic homeostasis in the regenerated intestines of *A. japonicus*.

Tissue regeneration after damage is an inflammation- and metabolism-demanding process [33]. This process of inflammation has three distinct phases: initially, the recruitment of pivotal inflammatory cells from the innate immune system is essential to trigger the reparative process; subsequently, pivotal inflammatory cells from the innate immune system switch to a repair state, which results in the proinflammatory cells beginning to subside; finally, the inflammatory cells of innate immunity response either apoptosis or exit the site of injury when tissue homeostasis is restored [21]. Recent studies showed that tissue regeneration is distinctly regulated by immunity response. Macrophages stimulated by IL-13 and/or IL-4 are significant sources of various growth factors, encompassing transforming growth factor-beta 1 (TGF-β1), insulin-like growth factor 1 (IGF-1), platelet-derived growth factor (PDGF), and vascular endothelial growth factor (VEGF) [34]. Intriguingly, our findings indicated that immune signaling pathways, including the NOD-like receptor (NLR) signaling pathway, the C-type lectin receptor signaling pathway, and the retinoic acid-inducible gene I (RIG-I)–like receptor signaling pathway, participate in the intestinal regeneration process following an 11-day of exposure to TBBPA. These outcomes suggest that the innate immune system is instrumental in tissue regenerative activities.

Superoxide dismutase (SOD) is the most important physiological antioxidant enzyme against lipid peroxidation and free radicals. Many findings have revealed that the activities of SOD can be affected by environmental toxins [26,28]. In this study, we found that the activities of SOD were increased in the intestinal regeneration process following an 11-day exposure to TBBPA, suggesting that lipid metabolic homeostasis is essential for tissue regeneration. Recent studies have also demonstrated that the imbalance of lipid metabolism could lead to many physiological processes disorders such as type II diabetes, obesity, and atherosclerosis. The metabolic homeostasis of the regenerated gut of sea cucumbers is affected when exposed to toxicants in the environment. In this study, we found that after 11 days of exposure to high levels of TBBPA, the homeostasis of lipid metabolism in the regenerated gut was dysfunctional in sea cucumbers.

The gut microbiome plays a wide range of roles in all aspects of host health. Intensive investigations have demonstrated that the gut microbiome affects tissue regeneration, intestinal permeability, metabolism, immune response, and even the development and occurrence of malignant tumors when the distribution and composition of the gut microbiome changes [35,36]. The gut microbiota can engage with the gut epithelium either directly via outer membrane vesicles released by Gram-negative bacteria, which may lead to alterations in intestinal stem cell (ISC) gene expression patterns, or indirectly through the bacterial metabolites that they secrete. Moreover, gut microbiota affects the healing and regeneration of intestinal epithelial cells, nerves, liver, and bone [8,9,10,11]. A recent study has demonstrated that regenerating gut tissues in sea cucumbers harbor a microbiome that potentially provides both energetic and immune benefits to the host. This includes microbes capable of fixing carbon and degrading harmful pathogens, highlighting the significant role of the gut microbiota in the intestinal regeneration process of sea cucumbers [37,38]. In addition, the distribution and composition of the gut microbiome of the regenerated intestine changed when it was exposed to toxins in the environment [6,39]. The results in this study found that the distribution and composition of the gut microbiome of the regenerated intestine, the thickness of the regenerated intestinal wall, and the villi length of the regenerated intestine was affected in sea cucumber after exposure to high levels of TBBPA.

Gene expression is modulated by trans-acting factors that bind to specific DNA elements, thereby recruiting RNA polymerases and additional cofactors essential for the initiation and elongation of transcription. These interactions occur at cis-acting elements located within promoter and enhancer regions [40]. The transcriptional underpinnings of tissue regeneration metabolism are governed by a cadre of transcription factors. These include ESRRα (estrogen-related receptor alpha), PPARα (peroxisome proliferator-activated receptor alpha), PPARγ (PPAR gamma), PGC-1α(PPARγ coactivator 1 alpha), the farnesoid X receptor, the pregnane X receptor, hepatocyte nuclear factor 4 alpha (HNF4α), and Kruppel-like factor 15 [40,41,42,43,44]. In general, any of these transcription factors loss reduced the expression level of the fatty acid metabolism–related genes. For instance, the process of metabolism after injury was regulated by a change from Kruppel-like factor 15 to KLF6 [43,45]. Likewise, the motifs for ESRRα, along with PPARα, HNF4A, and HNF1B, are enriched in the promoters of metabolic genes that are downregulated after injury [42]. Additionally, the expression of quinolinate phosphoribosyltransferase (QPRT), which is pivotal for NAD + metabolism, is under the cooperative regulation of PPARγ coactivator 1α and HNF4α [46]. These transcription factors also function as nuclear hormone receptors [47], suggesting that they may have overlapping roles in regulating metabolism and differentiation during tissue regeneration post-injury. The results of this study showed that there were 77 transcript factors with significantly different expression levels after exposure to TBBPA, suggested that TBBPA can induce an imbalance of metabolic homeostasis through regulating the expression level of transcription factors in the regenerated intestine of *A. japonicus*.

## 5. Conclusions

Our study revealed that the integrity, the intestinal wall thickness, and the villi length of the regenerated intestines decreased after exposure to high levels of TBBPA. An upregulation in the enzyme activity of superoxide dismutase (SOD) was observed, whereas the activity of lysozyme (LZM) remained unaffected following exposure to TBBPA. The diversity of microbial community in the regenerated intestines, as indicated by the relative abundance analysis, the principal coordinate analysis (PCoA), and non-metric multidimensional scaling (NMDS), was significantly influenced by TBBPA exposure. Furthermore, the metabolic homeostasis of the regenerated intestines was disrupted due to TBBPA exposure. Additionally, our data suggest that TBBPA can induce metabolic imbalances by modulating the expression levels of key transcription factors in the regenerated intestine of *A. japonicus*, underscoring the compound’s potential to disrupt normal physiological processes during tissue regeneration.

## Figures and Tables

**Table 1:** Percentage of intact regenerated gut morphologies of *A. japonicus* exposure to different concentrations of TBBPA.

	Intact	Partial	None	All	Percentage (%)
0 nmol/L	15	0	0	15	100
3 nmol/L	15	0	0	15	100
5 nmol/L	8	15	2	25	32
7 nmol/L	0	2	13	15	0

Note: Intact: The regeneration intestines were complete. Partial: The regeneration intestines were not complete. None: Regeneration intestine was not found. All: The number of dissected sea cucumbers. Percentage (%): Percentage of intact regenerated intestines.

**Table 2:** Summary of RNAseq data.

Samples ID.	Reads Number	Base Number	GC Content	Q30	Mapped Ratio
0 nmol/L-1	21,671,050	6,481,001,758	40.78%	94.83%	67.74%
0 nmol/L-2	19,270,474	5,764,867,558	40.66%	95.70%	68.25%
0 nmol/L-3	29,160,071	8,701,491,994	40.38%	97.59%	68.79%
3 nmol/L-1	22,879,225	6,829,701,852	40.63%	97.34%	68.68%
3 nmol/L-2	19,969,640	5,973,016,844	40.78%	95.84%	67.39%
3 nmol/L-3	21,896,070	6,548,816,556	40.88%	94.72%	68.05%
5 nmol/L-1	27,376,728	8,167,870,648	40.77%	97.84%	68.20%
5 nmol/L-2	26,586,205	7,932,849,934	40.21%	97.58%	68.63%
5 nmol/L-3	19,169,104	5,734,435,578	40.64%	95.32%	67.40%

## Data Availability

The raw data underpinning the findings of this manuscript have been deposited with the China National Center for Bioinformation (CNCB, https://www.cncb.ac.cn/?lang=en) under the accession numbers CRA020139 for the RNA-sequencing data and CRA020120 for the 16S rDNA sequencing data.
